# Quaternary cocrystals: combinatorial synthetic strategies based on long-range synthon Aufbau modules (LSAM)

**DOI:** 10.1107/S2052252515023957

**Published:** 2016-01-05

**Authors:** Ritesh Dubey, Niyaz A. Mir, Gautam R. Desiraju

**Affiliations:** a Solid State and Structural Chemistry Unit, Indian Institute of Science, Bangalore 560 012, India

**Keywords:** long-range synthon Aufbau module, crystal engineering, inermolecular interactions, polymorphism, cocrystals, crystal design

## Abstract

A synthetic strategy is outlined whereby a binary cocrystal may be developed in turn into a ternary and finally into a quaternary cocrystal. The strategy hinges on the concept of the long-range synthon Aufbau module (LSAM) which is a large supramolecular synthon containing more than one type of intermolecular interaction. Modulation of these interactions may be possible with the use of additional molecular components so that higher level cocrystals are produced. We report six quaternary cocrystals here. All are obtained as nearly exclusive crystallization products when four appropriate solid compounds are taken together in solution for crystallization.

## Abstract 2

A combinatorial synthetic approach is described for the isolation of quaternary cocrystals. The strategy outlines chemical and geometrical modulations in the long-range synthon Aufbau modules (LSAMs) to systematically increase the number of components.

## 1. Introduction   

The design of multicomponent molecular crystals is recognized as one of the challenging areas in modern crystal engineering (Aakeröy *et al.*, 2005 ▸; Tothadi & Desiraju, 2013 ▸; Tothadi *et al.*, 2011 ▸; Bolla & Nangia, 2015 ▸; Tothadi *et al.*, 2014 ▸). Any good synthetic scheme directed towards a higher cocrystal (ternary, quaternary) should ensure its appearance from a solution containing the various solid constituents in high yield, in other words, without two- or single-component byproducts (Dubey & Desiraju, 2014 ▸, 2015 ▸). The product should also be obtained in high supramolecular yield (Aakeröy *et al.*, 2001 ▸), in other words, it should be obtained without various polymorphs or compounds containing different synthons. In all cocrystals, there are favorable hetero-molecular interactions (Almarsson *et al.*, 2004 ▸; Desiraju *et al.*, 2011 ▸; Resnati *et al.*, 2015 ▸). The main difficulty in the design of higher component cocrystals is that it is very difficult to establish synthon hierarchies in systems where there are a large number of functionalities that can form numerous and diverse intermolecular interactions (Aakeröy *et al.*, 2005 ▸; Tothadi & Desiraju, 2013 ▸; Tothadi *et al.*, 2011 ▸, 2014 ▸; Bolla & Nangia, 2015 ▸). Out of this pool of competing interactions, it is necessary to avoid interaction crossover, or in other words certain supramolecular features need to be amplified at the expense of others (Aakeröy *et al.*, 2011 ▸; Saha *et al.*, 2005 ▸). Although supramolecular synthons have been invoked as retrosynthetic constructs to analyze and fashion crystal structures (Desiraju, 1995 ▸), simple synthon information is often insufficient to design real solids with tangible properties. As one moves to more complex and larger hydrogen-bonded architectures, the concept of the long range synthon Aufbau module (LSAM) may be more fruitful as these larger synthons contain more representative and characteristic information about symmetry, long range order and topology in the crystal structure (Ganguly & Desiraju, 2008 ▸, 2010 ▸). The concepts of the supramolecular synthon and the LSAM are not merely restricted to the understanding of crystal structures. Recent studies show that some of these smaller and larger synthons exist in solution prior to crystallization (Mukherjee *et al.*, 2014 ▸). The build-up of a crystal from individual molecules may well be considered as occurring through a smooth and systematic build-up in structural complexity. It is in this context that the extended synthon or LSAM becomes useful in crystal structure design.

Recently some of us (Dubey & Desiraju, 2014 ▸, 2015 ▸) have shown that the principles of constitutional dynamic chemistry (Lehn, 2013 ▸, 2015 ▸) may be applied to combinatorial crystal synthesis and that the process of building up of LSAMs in solution involves a selection of certain preferred molecular conformations and small synthons. In this view, there are libraries (Lehn, 1999 ▸) of real and virtual synthons in solution and an associated library of crystal structures which in themselves constitute a landscape (Thakur *et al.*, 2015*a* ▸). These ideas were demonstrated by us in the synthesis of ternary cocrystals of phloroglucinol (PGL) (Dubey & Desiraju, 2014 ▸) and the related polyhydroxy natural product, quercetin (Dubey & Desiraju, 2015 ▸). We showed that these polyhydric phenols form a large number of polymorphic and pseudopolymorphic binary cocrystals with ditopic bases like 1,2-bis(4-pyridyl)ethane (DPE), phenazine (PHE) and so on. These binaries lead to a much smaller number of ternaries when a selected third component was taken for co-crystallization. A small number of ternary cocrystals in a system where there are many binary possibilities showed that the binary to ternary progression is convergent; our results hinted that such convergence may be further exploited for the design of quaternary cocrystals. These ideas are illustrated in the present article. Implicit in this analysis is that stable large synthons persist in solution and that suitably selected compounds may be further attached to these LSAMs leading to higher component crystals. It is unnecessary and impractical to consider the design of every new crystal as an *ab initio* exercise (Dunitz, 2015 ▸; Thakur *et al.*, 2015*b* ▸; Lecomte *et al.*, 2015 ▸). Supramolecular synthons are kinetically favored crystallization intermediates and may be homologated to more elaborate structures, in solution, provided that the additional components are properly modulated in terms of intermolecular interactions (Desiraju, 2007 ▸).

## 2. Results and discussion   

**Figure 1:**
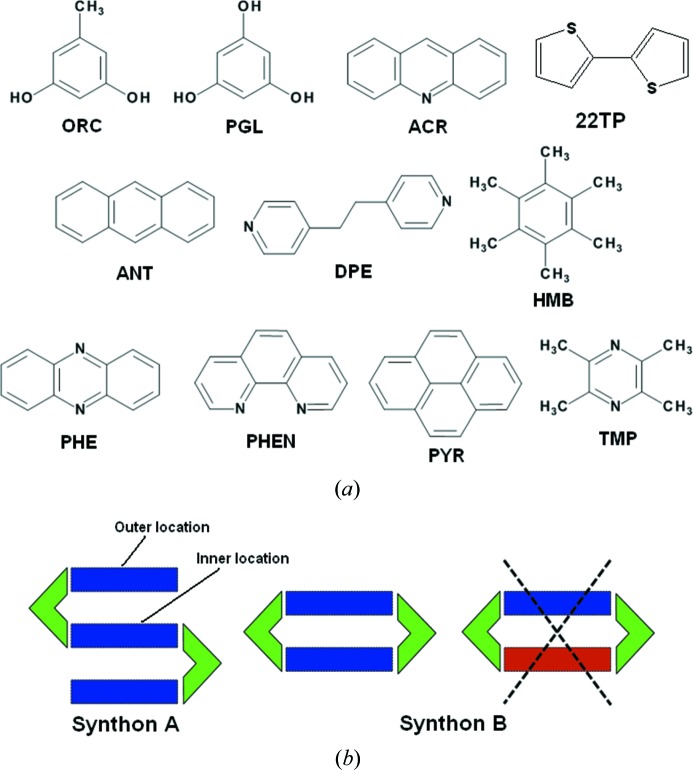
(*a*) Compounds in this study; (*b*) operating supramolecular synthons (**A** and **B**) in the crystal synthesis of multi-component systems. Polyhydric phenols ORC, PGL are shown in green. Blue, red are connector bases. Synthon **B** with different connector bases is not observed.

**Figure 2:**
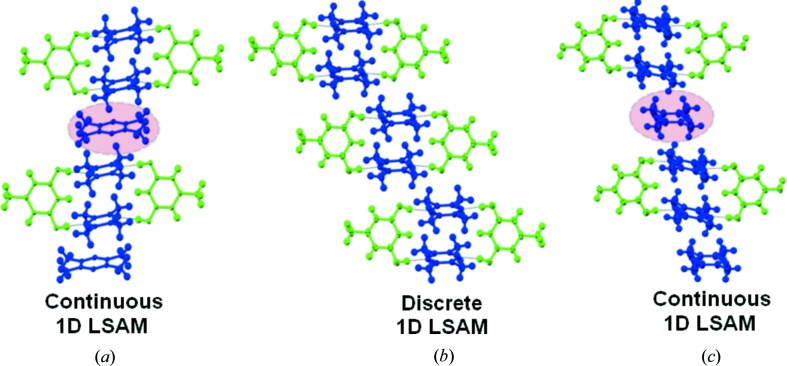
Binary cocrystals. (*a*) 2:3 ORC·TMP Form I; (*b*) 1:1 ORC·TMP Form II (hydrated); (*c*) 2:3 PGL·TMP. Distinct chemical species are color coded. Note the continuous one-dimensional LSAMs in ORC·TMP Form I and PGL·TMP and the different orientations of the pink-shaded TMP molecules in the two cases.

This article argues for the synthetic design of complex quaternary solids by systematic selection and fabrication from LSAMs in binary and ternary cocrystals. Fig. 1 ▸(*a*) shows a list of the compounds in this study with their acronyms, whilst Fig. 1 ▸(*b*) shows the relevant synthons (MacGillivray *et al.*, 2008 ▸). The starting points in this crystal engineering exercise are the binary O—H⋯N based cocrystals formed by the polyhydric phenols, orcinol (ORC) and phloroglucinol (PGL) with tetramethylpyrazine (TMP). Fig. 2 ▸ shows two crystal forms of ORC·TMP, and also the single form of 2:3 PGL·TMP. It is noted that each of the ORC·TMP forms (actually pseudopolymorphs) has a different LSAM. In one of them (2:3 Form I obtained from MeNO_2_), the TMP molecules form a more or less continuous one-dimensional array. This array is made up of closed TMP·ORC tetramers (synthon **B**, Fig. 2 ▸) and ‘free’ TMP molecules that are intercalated *via* C—H⋯π interactions. The second (1:1 Form II obtained from MeOH in an attempted ternary crystallization) consists of discrete synthon **B** modules that are laterally offset with respect to one another so that there are no C—H⋯π interactions. Fig. 2 ▸ also shows the structure of the 2:3 PGL·TMP binary and it may be seen that it takes a packing similar to Form I of ORC·TMP. We also note that the intercalated LSAM is capable of further structural modification: the ‘free’ TMP molecule could in principle be substituted with another flat aromatic molecule to yield a ternary (Tothadi *et al.*, 2011 ▸). However, the laterally offset LSAM is not capable of such extension. It has been suggested that LSAMs are generally one-dimensional in nature (Ganguly & Desiraju, 2010 ▸). In these prototype binary systems, the O—H⋯N and C—H⋯π interactions facilitate collinear molecular arrangements. Once this critical LSAM is identified, the next step is to fabricate it with optimized synthon hierarchies established during the landscape exploration to realise higher multi-component systems. Effectively, the one-dimensional LSAM is modular with respect to the three-dimensional structure, unlike the smaller synthons.

**Figure 3:**
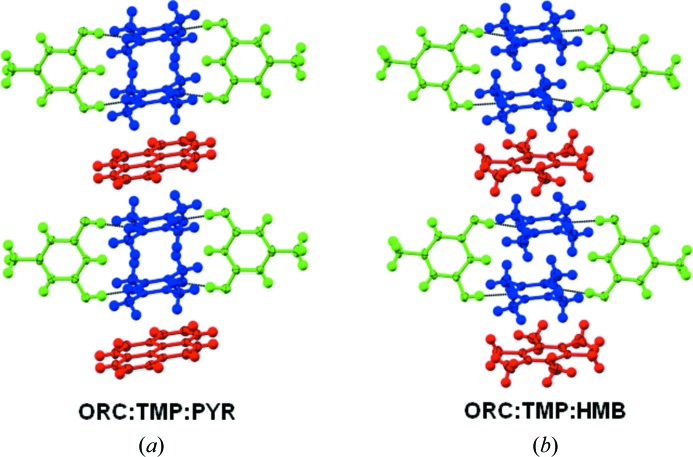
2:2:1 stoichiometric ternary cocrystals with supramolecular synthon **B**. (*a*) Orcinol-tetramethylpyrazine-pyrene; (*b*) orcinol-tetramethylpyrazine-hexamethylbenzene.

**Figure 4:**
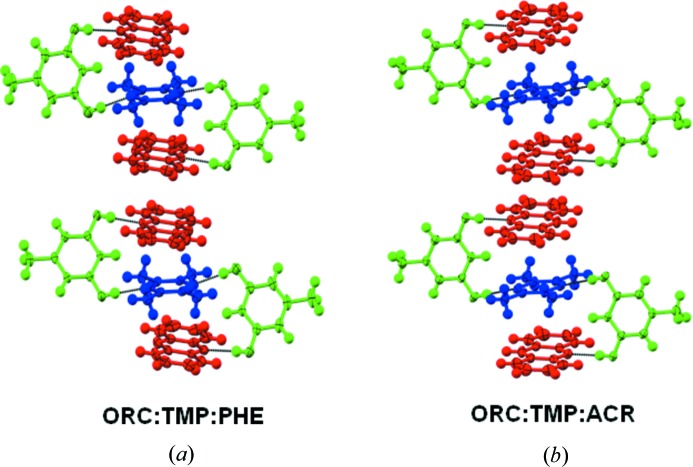
2:1:2 stoichiometricternary cocrystals with supramolecular synthon **A**. (*a*) Orcinol-tetramethylpyrazine-phenazine; (*b*) orcinol-tetramethylpyrazine-acridine.

Cocrystallization of a mixture of ORC and TMP with each of PHE, acridine (ACR), 1,10-phenanthroline (PHEN), 2,2-bisthiophene (22TP), hexamethylbenzene (HMB) and pyrene (PYR) results in stoichiometric ternary cocrystals. The structures of three of them (2:2:1 ORC·TMP·22TP, 2:2:1 ORC·TMP·HMB and 2:2:1 ORC·TMP·PYR) are along predicted lines and they may be considered as being obtained by substitution of the ‘free’ TMP molecule in Form I of the ORC·TMP binary with the new aromatic compound (Fig. 3 ▸). The other three structures (2:1:2 ORC·TMP·PHE, 2:1:2 ORC·TMP·ACR, 2:1:2 ORC·TMP·PHEN), however, are based on the open synthon **A** (Fig. 4 ▸) and the third compound is actually a part of this synthon. This third component is an electron-deficient species and stacks with itself leading to a one-dimensional array. The TMP molecule forms C—H⋯π interactions with the third component within synthon **A**. The fact that an ORC·TMP binary with open synthon **A** is not isolated indicates that this synthon is virtual (Dubey & Desiraju, 2014 ▸) with respect to the binary system but becomes accessible in the ternary system. A virtual synthon is defined as one which is the product of a potential molecular recognition event that does not take place in a certain system. In summary, the ORC·TMP system is quite adaptive: it can sustain continuous and discrete one-dimensional LSAMs; it can sustain open and closed O—H⋯N based synthons.

**Figure 5:**
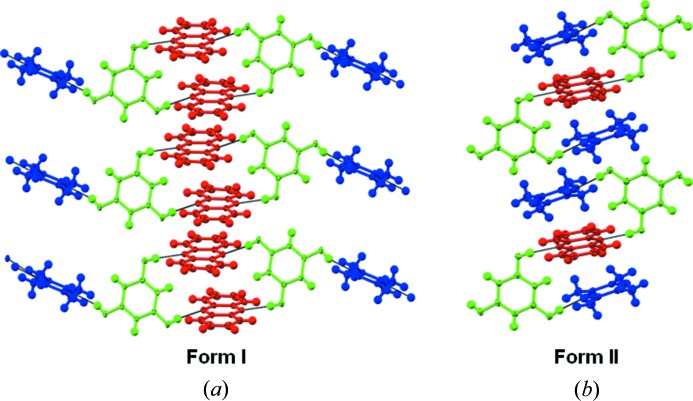
Pseudopolymorphs of the stoichiometric ternary cocrystal phloroglucinol-tetramethylpyrazine-phenazine. (*a*) 2:1:3 solid from MeCN; (*b*) 2:1:2 solid also obtained from MeCN.

Let us consider next the ternary cocrystals formed by PGL. Co-crystallization of equimolar amounts of PGL and TMP with each of PHE, PYR and DPE resulted in four structurally distinct ternaries. When PGL, TMP and PHE were cocrystallized, two forms were obtained depending on the solvent used. From MeCN we obtained the 2:1:3 Form I (Fig. 5 ▸) in which closed synthon **B** is constructed exclusively with PHE and the TMP molecules provide cross links *via* the third ‘hook’ hydroxy group of the PGL molecule. From MeCN also but under different conditions, we obtained the 2:1:2 Form II which is reminiscent of the ORC·TMP·PHE ternary except that PHE is in the ‘inner’ part of synthon **A** rather than in the ‘outer’ part. There was no contamination of either of these ternaries by the ‘other’ ternary in the crystallization experiments. *A priori*, it would not be possible to predict which structure one would obtain from MeCN under what conditions. What is important, however, is that there are a number of topologically similar crystal structures available to the system. Which one is actually obtained would seem to depend on the exact experimental conditions used. The system lends itself to high throughput methods. We maintain that we carried out a very large number of crystallization experiments on an entire array of compounds and solvent systems in a combinatorial manner. It is of interest to note that such examples of (pseudo)polymorphism are very rare in three component systems (Tothadi *et al.*, 2011 ▸).

**Figure 6:**
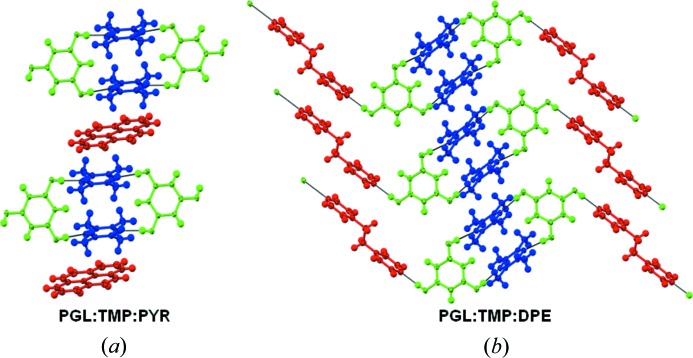
2:2:1 stoichiometric ternary cocrystals with supramolecular synthon **B**. (*a*) Phloroglucinol-tetramethylpyrazine-pyrene; (*b*) phloroglucinol-tetramethylpyrazine-1,2-bis(4-pyridyl)ethane.

**Figure 7:**
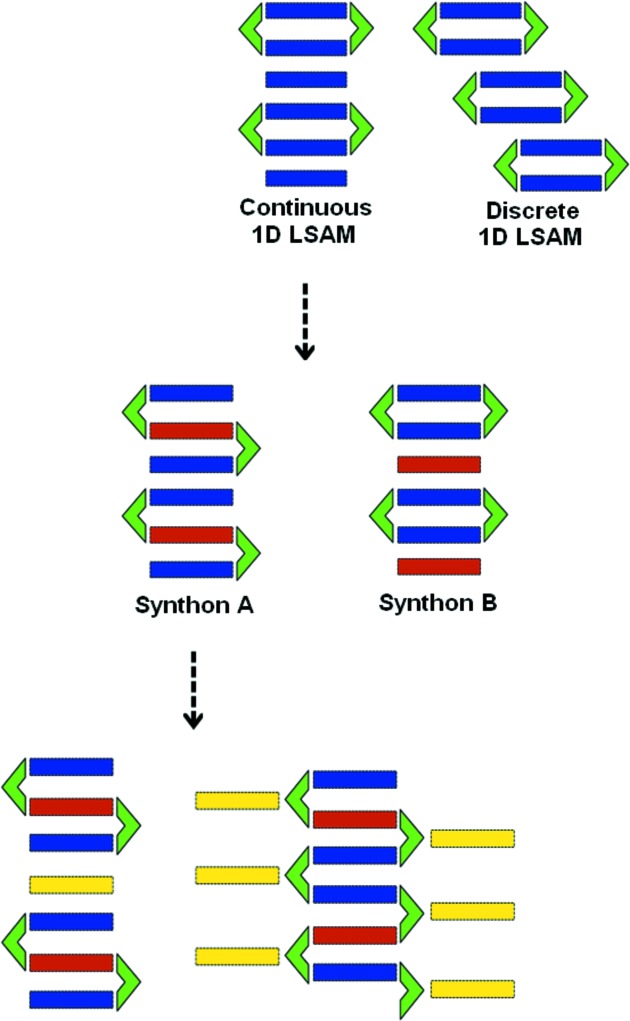
Schematic representation of the long range synthon Aufbau modules (LSAMs) in the crystal synthesis of multi-component crystals. Color coding signifies distinct chemical species in the LSAMs.

We now discuss the 2:2:1 PGL·TMP·PYR and 2:2:1 PGL·TMP·DPE ternary cocrystals (Fig. 6 ▸). The former is exactly like the synthon **B** based ternaries formed by ORC and TMP (with PYR, HMB and 22TP, Fig. 3 ▸). There is a clean replacement of the ‘free’ TMP in the ORC·TMP binary by the third compound. We note that the PGL·TMP binary does not contain any ‘free’ TMP once again showing synthon virtuality. The 2:2:1 PGL·TMP·DPE ternary cocrystal is similar to the 2:2:1 PGL·PHE·DPE ternary which we have already reported. Closed synthon **B** modules are cross linked with DPE through the third ‘hook’ hydroxy group of the PGL molecule. A study of the 10 ternary structures in this work shows that there are three regions which are capable of higher elaboration into quaternaries: open synthons of the type **A** can contain more than one heterocyclic base; open and closed synthons, **A** and **B**, can be intercalated/stacked with appropriate new planar molecules; the third ‘hook’ hydroxy group in PGL can be cross linked with a new component. We never observed a closed synthon **B** with two different heterocyclic bases and feel this to be an unlikely outcome (Fig. 1 ▸). These results open the way for isolation of quaternaries based on ORC and PGL. Fig. 7 ▸ is a flow chart of possible events. Because of synthon virtuality, it is not required that quaternaries in either of systems (ORC or PGL) must necessarily follow from ternaries in the same system. In the same way, a ternary may be based on a binary of the other phenol. All these structure types would appear to be isolable and which particular crystal form is isolated would seem to depend on experimental conditions of crystallization.

**Figure 8:**
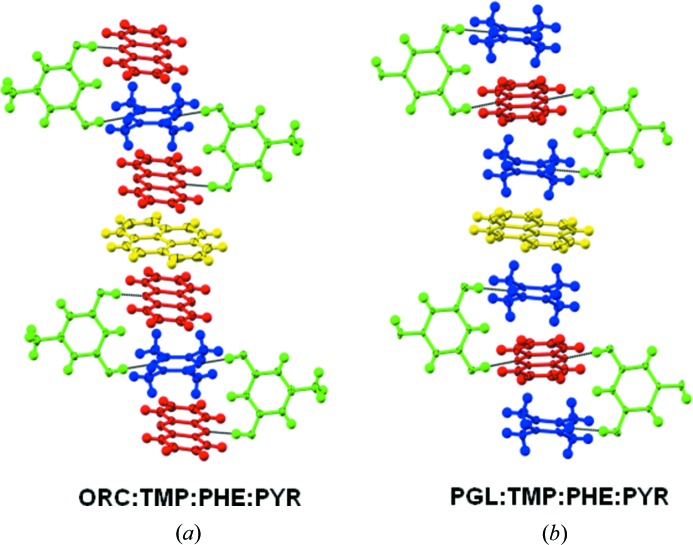
Stoichiometric quaternary cocrystals. (*a*) Orcinol-tetramethylpyrazine-phenazine-pyrene; (*b*) phloroglucinol-tetramethylpyrazine-phenazine-pyrene (obtained as a hydrate).

In practice, a total of six quaternaries were obtained, three each from ORC and PGL (Fig. 8 ▸ and section S3 of the supporting information). Let us consider these structures in turn. The 2:1:2:1 ORC·TMP·PHE·HMB structure follows smoothly from the ternary 2:1:2 ORC·TMP·PHE in a chemically reasonable manner. In the ternary, one observes π⋯π stacking between electron-deficient PHE molecules (3.29 Å, 3.54 Å). In the quaternary, HMB inserts in a classical donor–acceptor fashion (∼ 3.56 Å). Replacement of the electron-rich HMB by PYR and the ditopic PHE by ACR achieves the same result and one obtains the stoichiometric quaternaries 2:1:2:1 ORC·TMP·PHE·PYR and 2:1:2:1 ORC·TMP·ACR·PYR (see section S3). Coming next to PGL, it is not difficult to understand the crystal structure of the quaternary 2:1:1:1 PGL·TMP.PHE.DPE wherein an infinite synthon **A** based structure with two ditopic heterocycles is cross linked with DPE. The quaternaries 2:2:1:1 PGL.TMP.PHE.ANT and 2:2:1:1 PGL.TMP.PHE.PYR have very similar structures. Synthon **A** is constructed with TMP in the ‘outer’ locations and PHE in the ‘inner’ location. ANT and PYR intercalate with C—H⋯π interactions to give a columnar LSAM.

## 3. Conclusions   

The results obtained in this work validate the idea of using a supramolecular combinatorial library in the isolation of stoichiometric three- and four-component molecular crystals. We have earlier used this concept to make a single ternary cocrystal (Dubey & Desiraju, 2014 ▸). In the present study, 10 new ternaries have been reported. The crystal structures of these compounds contain extended synthon assemblies which are referred to as long-range synthon Aufbau modules or LSAMs. These LSAMs are modular units and new molecules can be appended or exchanged with molecules in the LSAM to obtain larger synthons that contain four distinct chemical entities, each of which forms a solid under ambient conditions in its native crystal structure. These larger units are the precursors to stoichiometric quaternary cocrystals, of which six are reported in this paper. While there is no spectroscopic evidence as yet for the existence of these LSAMs in solution (unlike in other cases already reported; Mukherjee *et al.*, 2014 ▸), the fact that there is so much fidelity between the ternary and quaternary crystal structures suggests the stability of the LSAMs in solution. We also note the degree of reversibility among synthons in solution. This leads to the concept of a virtual synthon, a supramolecular unit that may not lead to an isolable solid but manifests itself in a crystal structure of a higher-component solid. In other words, two compounds A and B may result in two putative synthons S_1_ and S_2_. While S_1_ may be found in a binary cocrystal, S_2_ may not be similarly found. However S_2_ (or a close equivalent) may be found in a ternary cocrystal between say, A, B and C. Similarly, a synthon that is virtual in a three-component system may be seen in a four-component cocrystal. This also implies that proof correction mechanisms exist in the crystallizations, perhaps leading to the specificity of outcome.^1^ It is impressive to note the specificity of these crystallizations in systems containing a large number of energetically similar intermolecular interactions. We found very little contamination of the crystalline products by other solids, whether they are of the same complexity level or whether they are simpler in nature. None of the quaternaries we isolated, save one, were contaminated for instance by polymorphic or pseudopolymorphic quaternaries or by ternaries and binaries. The key to using the LSAM concept in designing higher-component cocrystals seems to be a judicious choice of starting compounds, well balanced intermolecular interactions and high throughput methodologies of crystallization and crystallography, in other words a combinatorial approach.

## Supplementary Material

Crystal structure: contains datablock(s) global, BS_ORC_TMP, BS_ORC_TMP_Hydrate, BS_PGL_TMP.cif, QS_ORC_TMP_ACR_PYR, QS_ORC_TMP_PHE_HMB, QS_ORC_TMP_PHE_PYR, QS_PGL_TMP_PHE_ANT, QS_PGL_TMP_PHE_DPE, QS_PGL_TMP_PHE_PYR_hydrate, TS_ORC_TMP_22TP, TS_ORC_TMP_ACR, TS_ORC_TMP_HMB, TS_ORC_TMP_PHE, TS_ORC_TMP_PHEN, TS_ORC_TMP_PYR, TS_PGL_TMP_DPE, TS_PGL_TMP_PHE_Form_I, TS_PGL_TMP_PHE_Form_II, TS_PGL_TMP_PYR. DOI: 10.1107/S2052252515023957/hi5641sup1.cif

CIF files. DOI: 10.1107/S2052252515023957/hi5641sup3.zip

Supporting information. DOI: 10.1107/S2052252515023957/hi5641sup2.pdf

CCDC references: 1428091, 1428092, 1428093, 1428106, 1428104, 1428105, 1428107, 1428090, 1428108, 1428099, 1428095, 1428098, 1428094, 1428096, 1428097, 1428103, 1428100, 1428101, 1428102

## Footnotes

1 Proof correction is an essential feature in all crystallizations. It is especially important in complicated systems like MOFs and globular proteins, and it is achieved by virtue of the fact that the assembling interactions are relatively weak and the crystallization events are reversible. It is an important feature in the present sequence, which appears to be convergent in nature. See Feng *et al.* (2012 ▸).
