# Supplementary material for: Quaternary cocrystals: combinatorial synthetic strategies based on long-range synthon Aufbau modules (LSAM)
Source: IUCrJ. 2016 Jan 5;3(Pt 2):102–7. doi: 10.1107/S2052252515023957 (PMC4775158; doi:10.1107/S2052252515023957)

checkCIF/PLATON report

Structure factors have been supplied for datablock(s) shelx

THIS REPORT IS FOR GUIDANCE ONLY. IF USED AS PART OF A REVIEW PROCEDURE FOR PUBLICATION, IT SHOULD NOT REPLACE THE EXPERTISE OF AN EXPERIENCED CRYSTALLOGRAPHIC REFEREE.

No syntax errors found.   CIF dictionary   Interpreting this report

Datablock: shelx

Bond precision: C-C = 0.0023 A   Wavelength=0.71073

Cell:	a=8.876(4)	b=11.941(6)	c=17.980(9)
	alpha=90	beta=97.072(4)	gamma=90
Temperature:	150 K		

	Calculated	Reported
Volume	1891.2(16)	1891.2(16)
Space group	P 21/c	P 21/c
Hall group	-P 2ybc	-P 2ybc
Moiety formula	C16 H10, 2(C8 H12 N2), 2(C6 H6 O3)	?
Sum formula	C44 H46 N4 O6	C44 H46 N4 O6
Mr	726.85	726.85
Dx,g cm-3	1.276	1.276
Z	2	2
Mu (mm-1)	0.085	0.085
F000	772.0	772.0
F000’	772.34	
h,k,lmax	10,14,22	10,14,22
Nref	3718	3710
Tmin,Tmax	0.969,0.986	0.847,1.000
Tmin’	0.963	

Correction method= # Reported T Limits: Tmin=0.847 Tmax=1.000
AbsCorr = MULTI-SCAN

Data completeness= 0.998   Theta(max)= 25.997

R(reflections)= 0.0486(3403)   wR2(reflections)= 0.1530(3710)

S = 1.104   Npar= 251

The following ALERTS were generated. Each ALERT has the format
test-name_ALERT_alert-type_alert-level.
Click on the hyperlinks for more details of the test.

Alert level B

```
PLAT919_ALERT_3_B Reflection # Likely Affected by the Beamstop ...          2 Check
PLAT934_ALERT_3_B Number of (Iobs-Icalc)/SigmaW > 10 Outliers ....          2 Check
PLAT939_ALERT_3_B Large Value of Not (SHELXL) Weight Optimized S .     215.85
```

Alert level C

```
PLAT906_ALERT_3_C Large K value in the Analysis of Variance ......      2.797 Check
PLAT911_ALERT_3_C Missing # FCF Refl Between THmin & STh/L=  0.600          8 Report
PLAT918_ALERT_3_C Reflection(s) with I(obs) much smaller I(calc) .          3 Check
```

Alert level G

```
PLAT007_ALERT_5_G Number of Unrefined Donor-H Atoms ..............          3 Report
PLAT333_ALERT_2_G Check Large Av C6-Ring C-C Dist. C15    -C22_a         1.42 Ang.
PLAT333_ALERT_2_G Check Large Av C6-Ring C-C Dist. C20    -C22           1.42 Ang.
PLAT961_ALERT_5_G Dataset Contains no Negative Intensities .......     Please Check
```

---

0 **ALERT level A** = Most likely a serious problem - resolve or explain
3 **ALERT level B** = A potentially serious problem, consider carefully
3 **ALERT level C** = Check. Ensure it is not caused by an omission or oversight
4 **ALERT level G** = General information/check it is not something unexpected

0 ALERT type 1 CIF construction/syntax error, inconsistent or missing data
2 ALERT type 2 Indicator that the structure model may be wrong or deficient
6 ALERT type 3 Indicator that the structure quality may be low
0 ALERT type 4 Improvement, methodology, query or suggestion
2 ALERT type 5 Informative message, check

It is advisable to attempt to resolve as many as possible of the alerts in all categories. Often the minor alerts point to easily fixed oversights, errors and omissions in your CIF or refinement strategy, so attention to these fine details can be worthwhile. In order to resolve some of the more serious problems it may be necessary to carry out additional measurements or structure refinements. However, the purpose of your study may justify the reported deviations and the more serious of these should normally be commented upon in the discussion or experimental section of a paper or in the "special_details" fields of the CIF. checkCIF was carefully designed to identify outliers and unusual parameters, but every test has its limitations and alerts that are not important in a particular case may appear. Conversely, the absence of alerts does not guarantee there are no aspects of the results needing attention. It is up to the individual to critically assess their own results and, if necessary, seek expert advice.

Publication of your CIF in IUCr journals

A basic structural check has been run on your CIF. These basic checks will be run on all CIFs submitted for publication in IUCr journals (*Acta Crystallographica*, *Journal of Applied Crystallography*, *Journal of Synchrotron Radiation*); however, if you intend to submit to *Acta Crystallographica Section C* or *E*, you should make sure that full publication checks are run on the final version of your CIF prior to submission.

Publication of your CIF in other journals

Please refer to the *Notes for Authors* of the relevant journal for any special instructions relating to CIF submission.

PLATON version of 21/06/2015; check.def file version of 21/06/2015

Datablock shelx - ellipsoid plot

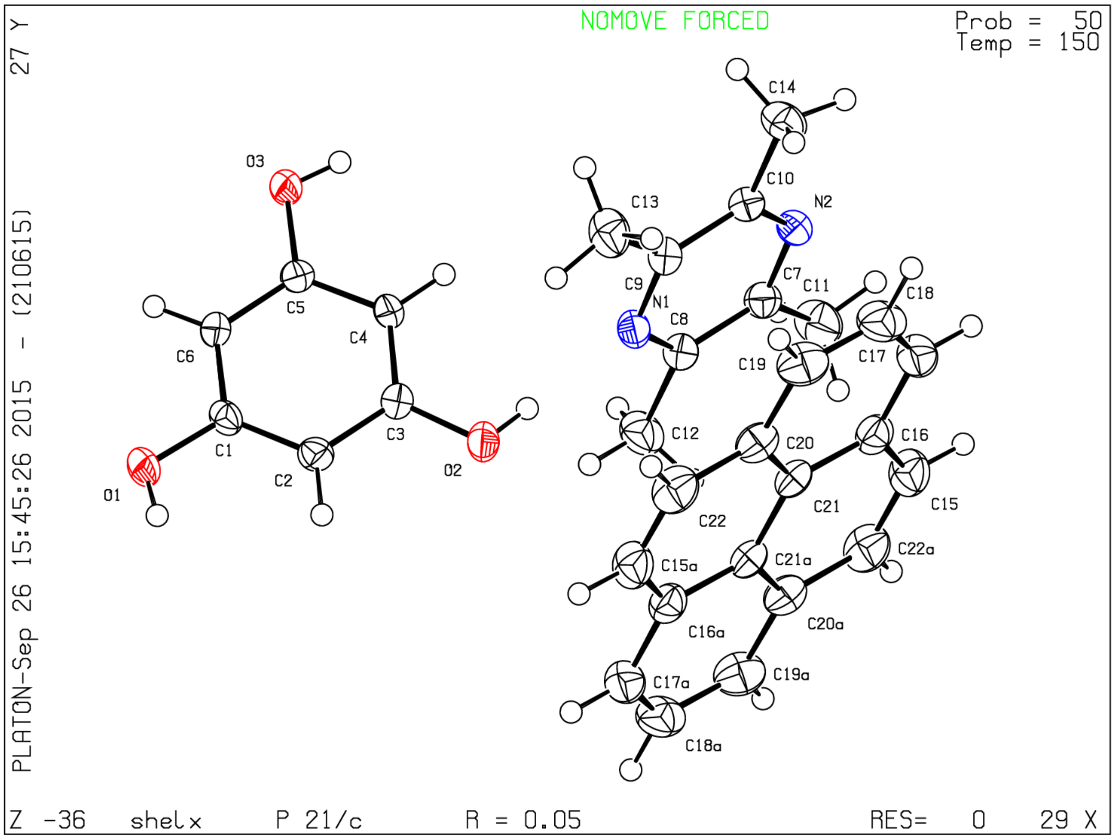

Supplement: Supplementary file 2 [file m-03-00102-sup3.zip › Checkcif Folder/12. TS PGL TMP PYR.pdf]
