# Supplementary material for: Quaternary cocrystals: combinatorial synthetic strategies based on long-range synthon Aufbau modules (LSAM)
Source: IUCrJ. 2016 Jan 5;3(Pt 2):102–7. doi: 10.1107/S2052252515023957 (PMC4775158; doi:10.1107/S2052252515023957)

checkCIF/PLATON report

You have not supplied any structure factors. As a result the full set of tests cannot be run.

THIS REPORT IS FOR GUIDANCE ONLY. IF USED AS PART OF A REVIEW PROCEDURE FOR PUBLICATION, IT SHOULD NOT REPLACE THE EXPERTISE OF AN EXPERIENCED CRYSTALLOGRAPHIC REFEREE.

No syntax errors found.   CIF dictionary   Interpreting this report

Datablock: shelx

No errors found in this datablock

```
Bond precision:  C-C = 0.0026 A          Wavelength=0.71073

Cell:           a=8.9943(15)      b=9.5169(16)     c=14.927(3)
                alpha=74.162(5)   beta=77.589(5)   gamma=89.115(6)
Temperature:    150 K
```

	Calculated	Reported
Volume	1199.2(4)	1199.2(4)
Space group	P -1	P -1
Hall group	-P 1	-P 1
Moiety formula	C12 H8 N2, 0.5(C12 H18), 0.5(C8 H12 N2), C7 H8 O2	?
Sum formula	C29 H31 N3 O2	C29 H31 N3 O2
Mr	453.57	453.57
Dx,g cm-3	1.256	1.256
Z	2	2
Mu (mm-1)	0.079	0.079
F000	484.0	484.0
F000’	484.19	
h,k,lmax	11,12,19	11,12,19
Nref	5503	5486
Tmin,Tmax	0.984,0.988	0.796,1.000
Tmin’	0.984	

```
Correction method= # Reported T Limits: Tmin=0.796 Tmax=1.000
AbsCorr = MULTI-SCAN

Data completeness= 0.997          Theta(max)= 27.484

R(reflections)= 0.0555( 3925)     wR2(reflections)= 0.1537( 5486)

S = 1.053                  Npar= 321
```

It is advisable to attempt to resolve as many as possible of the alerts in all categories. Often the minor alerts point to easily fixed oversights, errors and omissions in your CIF or refinement strategy, so attention to these fine details can be worthwhile. In order to resolve some of the more serious problems it may be necessary to carry out additional measurements or structure refinements. However, the purpose of your study may justify the reported deviations and the more serious of these should normally be commented upon in the discussion or experimental section of a paper or in the "special_details" fields of the CIF. checkCIF was carefully designed to identify outliers and unusual parameters, but every test has its limitations and alerts that are not important in a particular case may appear. Conversely, the absence of alerts does not guarantee there are no aspects of the results needing attention. It is up to the individual to critically assess their own results and, if necessary, seek expert advice.

Publication of your CIF in IUCr journals

A basic structural check has been run on your CIF. These basic checks will be run on all CIFs submitted for publication in IUCr journals (*Acta Crystallographica*, *Journal of Applied Crystallography*, *Journal of Synchrotron Radiation*); however, if you intend to submit to *Acta Crystallographica Section C* or *E*, you should make sure that full publication checks are run on the final version of your CIF prior to submission.

Publication of your CIF in other journals

Please refer to the *Notes for Authors* of the relevant journal for any special instructions relating to CIF submission.

PLATON version of 21/06/2015; check.def file version of 21/06/2015

Datablock shelx - ellipsoid plot

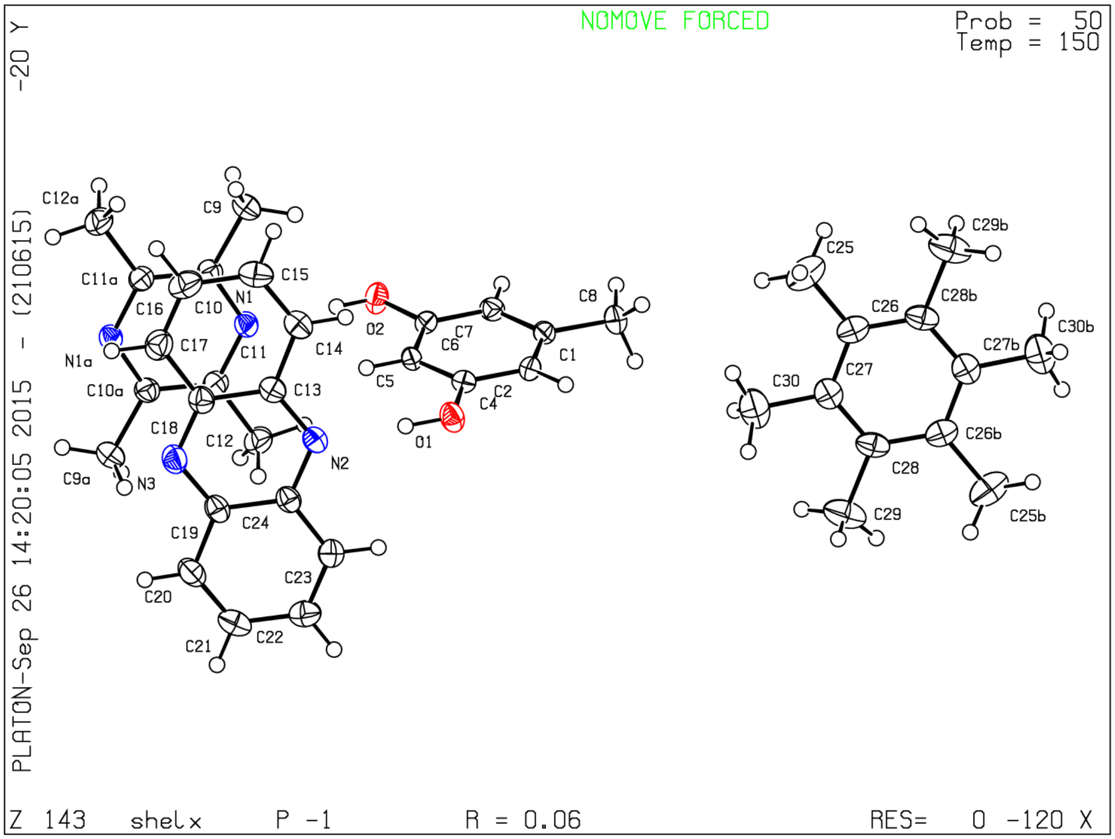

Supplement: Supplementary file 2 [file m-03-00102-sup3.zip › Checkcif Folder/14. QS ORC TMP PHE HMB.pdf]
