# Supplementary material for: Quaternary cocrystals: combinatorial synthetic strategies based on long-range synthon Aufbau modules (LSAM)
Source: IUCrJ. 2016 Jan 5;3(Pt 2):102–7. doi: 10.1107/S2052252515023957 (PMC4775158; doi:10.1107/S2052252515023957)

checkCIF/PLATON report

You have not supplied any structure factors. As a result the full set of tests cannot be run.

THIS REPORT IS FOR GUIDANCE ONLY. IF USED AS PART OF A REVIEW PROCEDURE FOR PUBLICATION, IT SHOULD NOT REPLACE THE EXPERTISE OF AN EXPERIENCED CRYSTALLOGRAPHIC REFEREE.

No syntax errors found. CIF dictionary Interpreting this report

Datablock: shelx

```
Bond precision:  C-C = 0.0100 A          Wavelength=0.71073

Cell:           a=10.1510(11)      b=11.2084(12)     c=12.3363(13)
                alpha=102.012(3)   beta=105.153(3)   gamma=112.368(3)
Temperature:    150 K

                  Calculated                  Reported
Volume            1176.1(2)                   1176.1(2)
Space group       P -1                        P -1
Hall group        -P 1                        -P 1
                  C16 H10, C12 H8 N2, 2(C8
Moiety formula    H12 N2), 2(C6 H6 O3), 2(H   ?
                  O)
Sum formula       C56 H56 N6 O8               C56 H56 N6 O8
Mr                941.07                      941.06
Dx,g cm-3         1.329                       1.329
Z                 1                           1
Mu (mm-1)         0.090                       0.090
F000              498.0                       498.0
F000'             498.22
h,k,lmax          12,13,15                    12,13,15
Nref              4627                        4600
Tmin,Tmax         0.955,0.980                 0.955,0.980
Tmin'             0.935

Correction method= # Reported T Limits: Tmin=0.955 Tmax=0.980
AbsCorr = ANALYTICAL

Data completeness= 0.994          Theta(max)= 25.999

R(reflections)= 0.1045( 3988)     wR2(reflections)= 0.4122( 4600)

S = 1.193                   Npar= 327
```

The following ALERTS were generated. Each ALERT has the format
      test-name_ALERT_alert-type_alert-level.
Click on the hyperlinks for more details of the test.

Alert level B

```
DIFMX01_ALERT_2_B  The maximum difference density is > 0.1*ZMAX*1.00
            _refine_diff_density_max given =      1.115
            Test value =      0.800
RFACR01_ALERT_3_B  The value of the weighted R factor is > 0.35
            Weighted R factor given   0.412
PLAT084_ALERT_3_B High wR2 Value (i.e. > 0.25) ...................       0.41 Report
PLAT097_ALERT_2_B Large Reported Max.  (Positive) Residual Density       1.12 eA-3
PLAT355_ALERT_3_B Long    O-H (X0.82,N0.98A)  O4     -   H4A    ..       1.11 Ang.
PLAT417_ALERT_2_B Short Inter D-H..H-D       H1     ..  H4A     ..       2.02 Ang.
```

Alert level C

```
DIFMN02_ALERT_2_C  The minimum difference density is < -0.1*ZMAX*0.75
            _refine_diff_density_min given =     -0.605
            Test value =     -0.600
DIFMN03_ALERT_1_C  The minimum difference density is < -0.1*ZMAX*0.75
            The relevant atom site should be identified.
DIFMX02_ALERT_1_C  The maximum difference density is > 0.1*ZMAX*0.75
            The relevant atom site should be identified.
RFACG01_ALERT_3_C  The value of the R factor is > 0.10
            R factor given   0.105
PLAT098_ALERT_2_C Large Reported Min.  (Negative) Residual Density      -0.61 eA-3
PLAT250_ALERT_2_C Large U3/U1 Ratio for Average U(i,j) Tensor ....        2.5 Note
PLAT340_ALERT_3_C Low Bond Precision on  C-C Bonds ...............     0.0100 Ang.
PLAT790_ALERT_4_C Centre of Gravity not Within Unit Cell: Resd.  #          1 Note
              C16 H10
```

Alert level G

```
PLAT007_ALERT_5_G Number of Unrefined Donor-H Atoms ..............          3 Report
PLAT063_ALERT_4_G Crystal Size Likely too Large for Beam Size ....       0.75 mm
PLAT066_ALERT_1_G Predicted and Reported Tmin&Tmax Range Identical          ? Check
PLAT072_ALERT_2_G SHELXL First  Parameter in WGHT Unusually Large.       0.15 Report
PLAT083_ALERT_2_G SHELXL Second Parameter in WGHT Unusually Large.      12.41 Why ?
PLAT154_ALERT_1_G The su’s on the Cell Angles are Equal ..........    0.00300 Degree
PLAT790_ALERT_4_G Centre of Gravity not Within Unit Cell: Resd.  #          5 Note
              H O
```

---

0 **ALERT level A** = Most likely a serious problem - resolve or explain
6 **ALERT level B** = A potentially serious problem, consider carefully
8 **ALERT level C** = Check. Ensure it is not caused by an omission or oversight
7 **ALERT level G** = General information/check it is not something unexpected

4 ALERT type 1 CIF construction/syntax error, inconsistent or missing data
8 ALERT type 2 Indicator that the structure model may be wrong or deficient
5 ALERT type 3 Indicator that the structure quality may be low
3 ALERT type 4 Improvement, methodology, query or suggestion
1 ALERT type 5 Informative message, check

It is advisable to attempt to resolve as many as possible of the alerts in all categories. Often the minor alerts point to easily fixed oversights, errors and omissions in your CIF or refinement strategy, so attention to these fine details can be worthwhile. In order to resolve some of the more serious problems it may be necessary to carry out additional measurements or structure refinements. However, the purpose of your study may justify the reported deviations and the more serious of these should normally be commented upon in the discussion or experimental section of a paper or in the "special_details" fields of the CIF. checkCIF was carefully designed to identify outliers and unusual parameters, but every test has its limitations and alerts that are not important in a particular case may appear. Conversely, the absence of alerts does not guarantee there are no aspects of the results needing attention. It is up to the individual to critically assess their own results and, if necessary, seek expert advice.

Publication of your CIF in IUCr journals

A basic structural check has been run on your CIF. These basic checks will be run on all CIFs submitted for publication in IUCr journals (*Acta Crystallographica*, *Journal of Applied Crystallography*, *Journal of Synchrotron Radiation*); however, if you intend to submit to *Acta Crystallographica Section C* or *E*, you should make sure that full publication checks are run on the final version of your CIF prior to submission.

Publication of your CIF in other journals

Please refer to the *Notes for Authors* of the relevant journal for any special instructions relating to CIF submission.

PLATON version of 21/06/2015; check.def file version of 21/06/2015

Datablock shelx - ellipsoid plot

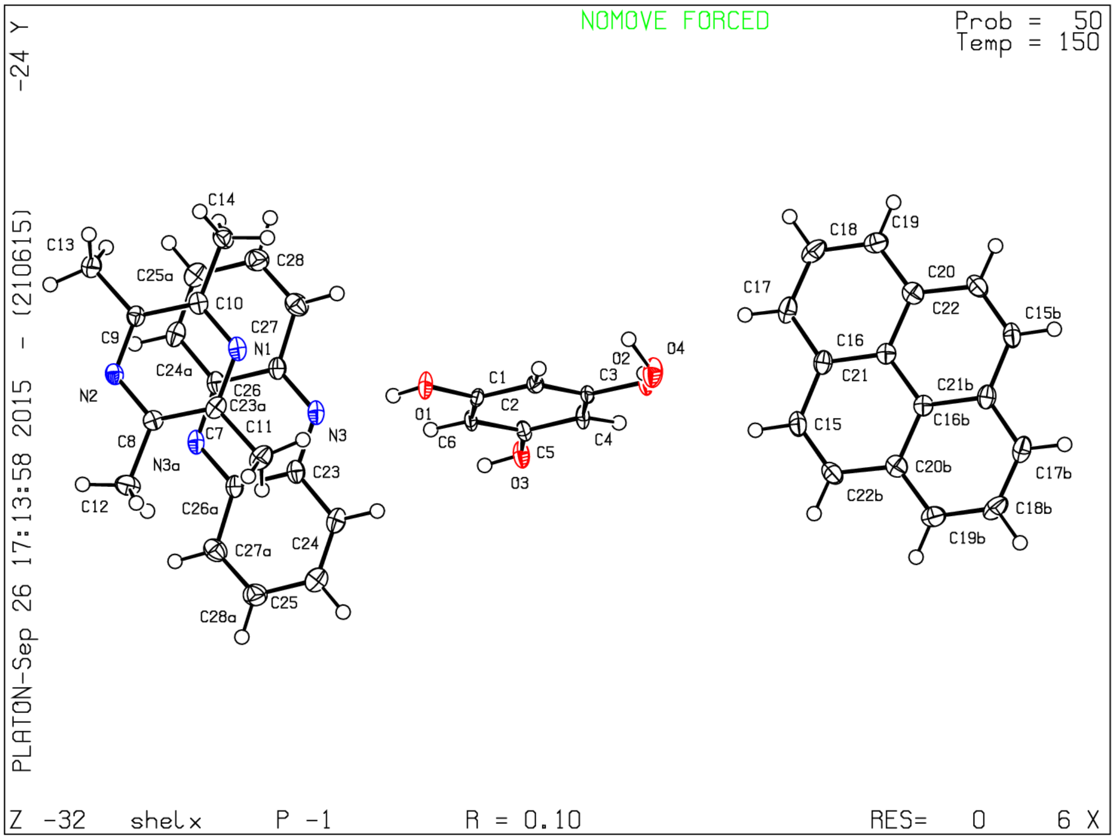

Supplement: Supplementary file 2 [file m-03-00102-sup3.zip › Checkcif Folder/18. QS PGL TMP PHE PYR hydrate.pdf]
