# Supplementary material for: Quaternary cocrystals: combinatorial synthetic strategies based on long-range synthon Aufbau modules (LSAM)
Source: IUCrJ. 2016 Jan 5;3(Pt 2):102–7. doi: 10.1107/S2052252515023957 (PMC4775158; doi:10.1107/S2052252515023957)
Supplement: Supplementary file 3 [file m-03-00102-sup2.pdf]

Supporting information

Quaternary cocrystals. Combinatorial synthetic strategies based on Long-range Synthon Aufbau Modules (LSAM)

Ritesh Dubey, Niyaz A. Mir, Gautam R. Desiraju*
Solid State and Structural Chemistry Unit,
Indian Institute of Science, Bangalore 560 012
desiraju@sscu.iisc.ernet.in

Contents

S1: Characterization techniques

S2: Crystallization details

S3: Quaternary cocrystals with orcinol (ORC) and phloroglucinol (PGL)

S4: Crystallographic tables

S1. Characterization techniques

Single crystal x-ray data for all crystals were collected on a *Rigaku Mercury 375/M CCD (XtaLAB mini)* diffractometer using graphite monochromator Mo-Kα radiation at 150 K and were processed with *Rigaku crystal clear software.* Some of the datasets (**3**, **17**, **18**)were collected on a *Bruker D8 Quest diffractometer* equipped with Oxford cryosystems N_2 open-flow cryostat using *MoKα* radiation. Data integration and data reduction were carried out using the SAINTPLUS program. Structure solution and refinement of all crystal structures were performed using SHELX-2013[1] embedded in the WinGX suite.[2]All non-hydrogen atoms were refined anisotropically by full-matrix least-squares method. Hydrogen atoms were fixed on riding model but some of the acidic hydrogen atoms were located via Fourier maps. Mercury version 3.5 was used for molecular representations and packing diagrams.

1. G. M. Sheldrick, *ActaCrystallogr., Sect. C,* 2015, **71**, 3.
2. L. J. Farrugia, *J. Appl. Crystallogr.* 1999, **32**, 837.

S2. Crystallization Details:

Solvent assisted grinding procedure was employed for crystallization. The solid components to be crystallized are taken together, in definite stoichiometric ratios, in a mortar along with a few drops of a solvent. The mixture is then ground with a pestle and the process is repeated 2-3 times to get a homogenous mixture. The solid mixture is then kept for crystallizations in different solvents.

a) *Orcinol.Tetramethylpyrazine* **(1)**: Orcinol and tetramethylpyrazine were taken in 1:2 molar ratio and ground in a mortar with a pestle for ten minutes along with 2-3 drops of methanol. Colorless block shaped diffraction quality crystals were obtained from nitromethane after 3-4 days in desiccator.

b) *Orcinol.Tetramethylpyrazinehydrate* **(2)**: Orcinol, tetramethylpyrazine and 4-cyanopyridine were taken in 2:1:1 molar ratio and ground in a mortar with a pestle for ten minutes along with 2-3 drops of methanol. Light brown colored block shaped diffraction quality crystals were obtained from methanol after 3-4 days.

c) *Phloroglucinol.Tetramethylpyrazine* **(3)**: Phloroglucinol and tetramethylpyrazine were taken in 1:1 molar ratio and ground in a mortar with a pestle for ten minutes along with 2-3 drops of tetrahydrofuran (THF). Colorless block shaped diffraction quality crystals were obtained from ethyl acetate after 3-4 days.

d) *Orcinol.Tetramethylpyrazine.Phenazine* **(4)**: A 2:1:1 molar ratio of orcinol, tetramethylpyrazine and phenazine were taken in a mortar and ground with a pestle for ten minutes along with 2-3 drops of methanol. Yellow colored block shaped diffraction quality crystals were obtained from nitromethane after 3-4 days.

e) *Orcinol.Tetramethylpyrazine.Acridine* **(5)**: A 2:1:1 molar ratio of orcinol, tetramethylpyrazine and acridine were taken in a mortar and ground with a pestle for ten minutes along with 2-3 drops of methanol. Colorless block shaped diffraction quality crystals were obtained from chloroform after 3-4 days.

f) *Orcinol.Tetramethylpyrazine.1,10-Phenanthroline* **(6)**: Orcinol, tetramethylpyrazine and *1,10-phenanthroline* were taken in 2:1:1 molar ratio along with 2-3 drops of methanol and ground in a mortar with a pestle for ten minutes. Light brown colored block shaped diffraction quality crystals were obtained from nitromethane after 3-4 days.

g) *Orcinol.Tetramethylpyrazine.Pyrene* **(7)**: Orcinol, tetramethylpyrazine, acridine and pyrene were taken in 2:2:2:1 molar ratio along with 2-3 drops of methanol and ground in a mortar with a pestle for ten minutes. Yellow colored block shaped diffraction quality crystals were obtained from tetrahydrofuran after 3-4 days.

h) *Orcinol.Tetramethylpyrazine.Hexamethylbenzene* **(8)**: Orcinol, tetramethylpyrazine and hexamethylbenzene were taken in 2:2:1 molar ratio and ground in a mortar with a pestle for ten minutes along with 2-3 drops of methanol. Colorless block shaped diffraction quality crystals were obtained from nitromethane after 3-4 days.

i) Orcinol.Ttetramethylpyrazine.2,2'-Bisthiophene **(9)**:A 2:1:1 molar ratio of orcinol, tetramethylpyrazine and *2,2'-bisthiophene* were taken along with 2-3 drops of methanol in a mortar and ground with a pestle for ten minutes. Colorless block shaped diffraction quality crystals were obtained from THF after 3-4 days.

j) *Phloroglucinol.Tetramethylpyrazine.Phenazine (form I)* **(10)**:An equimolar mixture of phloroglucinol, tetramethylpyrazine and phenazine were taken in a mortar. Few drops of THF were added and the mixture was ground with a pestle for ten minutes. Orange colored block shaped diffraction quality crystals were obtained at $5^{o}C$ from acetonitrile after 4-5 days.

k) *Phloroglucinol.Tetramethylpyrazine.Phenazine (form II)* **(11)**: A 2:2:2:1 mixture of phloroglucinol, tetramethylpyrazine, phenazine and anthracene along with 2-3 drops of THF was ground in a mortar with a pestle for ten minutes. Orange colored block shaped diffraction quality crystals were obtained concomitantly with **17** from acetonitrile at $5^{o}C$ after 4-5 days.

l) *Phloroglucinol.Tetramethylpyrazine.Pyrene* **(12)**:A 1:1:1 mixture of phloroglucinol, tetramethylpyrazine and pyrene along with 2-3 drops of THF was ground in a mortar with a pestle for ten minutes. Orange colored block shaped diffraction quality crystals were obtained from nitromethane after 3-4 days.

m) *Phloroglucinol.Tetramethylpyrazine.1,2-Bis(4-pyridyl)ethane* **(13)***:* An equimolar mixture of phloroglucinol, tetramethylpyrazine and 1,2-*bis*(4-*pyridyl*)*ethane* along with 2-3 drops of THF was ground in a mortar with a pestle for ten minutes. Orange colored block shaped diffraction quality crystals were obtained from nitromethane after 3-4 days.

n) *Orcinol.Tetramethylpyrazine.Phenazine.Hexamethylbenzene* **(14)**: Orcinol, tetramethylpyrazine, phenazine and hexamethylbenzene were taken in 2:2:2:1 molar ratio along with 2-3 drops of methanol and ground in a mortar with a pestle for ten minutes. Yellow colored block shaped diffraction quality crystals were obtained from nitromethane after 3-4 days.

o) *Orcinol.Tetramethylpyrazine.Phenazine.Pyrene* **(15)**: Orcinol, tetramethylpyrazine, phenazine and pyrene were taken in 2:2:2:1 molar ratio and ground in a mortar with a pestle for ten minutes along with 2-3 drops of methanol. Yellow colored block shaped diffraction quality crystals were obtained from acetonitrile after 3-4 days.

p) *Orcinol.Tetetramethylpyrazine.Acridine.Pyrene* **(16)**: Orcinol, tetramethylpyrazine, acridine and pyrene were taken in 2:2:2:1 molar ratio and ground in a mortar with a pestle for ten minutes along with 2-3 drops of methanol. Light yellow colored block shaped diffraction quality crystals were obtained from 1,4-dioxane after 3-4 days.

q) *Phloroglucinol.Tetramethylpyrazine.Phenazine.Anthracene* **(17)**: A 2:2:2:1 mixture of phloroglucinol, tetramethylpyrazine, phenazine and anthracene along with 2-3 drops of THF was ground in a mortar with a pestle for ten minutes. Red colored block shaped diffraction quality crystals were obtained concomitantly along with **11**from acetonitrile at 5°C after 4-5 days.

r) *Phloroglucinol.Tetramethylpyrazine.Phenazine.Pyrenehydrate* **(18)**: A 2:2:2:1 mixture of phloroglucinol, tetramethylpyrazine, phenazine and pyrene along with 2-3 drops of THF was ground in a mortar with a pestle for ten minutes. Orange colored block shaped diffraction quality crystals were obtained from a 1:1 mixture of benzene and nitromethane after 3-4 days.

s) *Phloroglucinol.Tetramethylpyrazine.Phenazine.1,2-Bis(4-pyridyl)ethane* **(19)***:* An equimolar mixture of phloroglucinol, tetramethylpyrazine, phenazine and 1,2-*bis*(4-pyridyl)ethane were taken in a mortar and the mixture was ground, along with few drops of THF, for about ten minutes. Orange colored block shaped diffraction quality crystals were obtained from acetonitrile after 3-4 days.

S3. Quaternary cocrystals

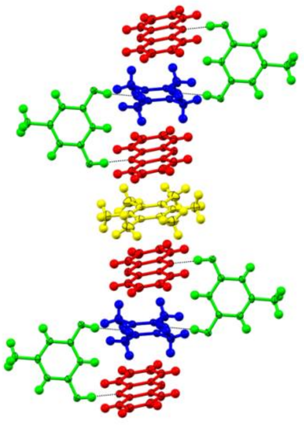

ORC.TMP.PHE.HMB

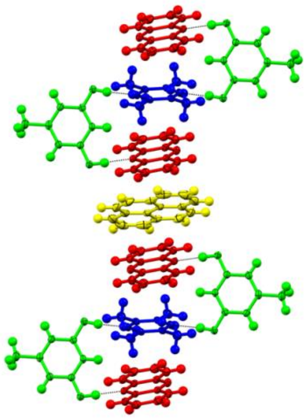

ORC.TMP.PHE.PYR

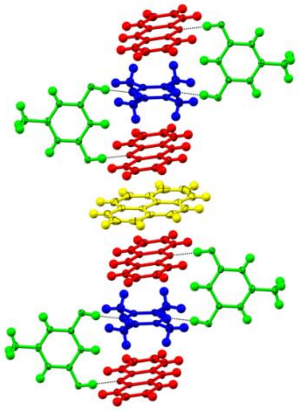

ORC.TMP.ACR.PYR

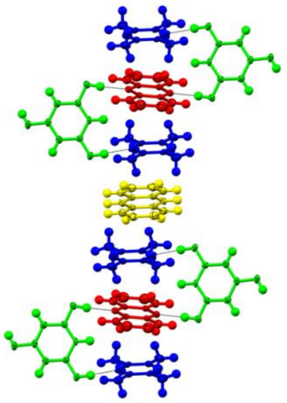

PGL.TMP.PHE.ANT

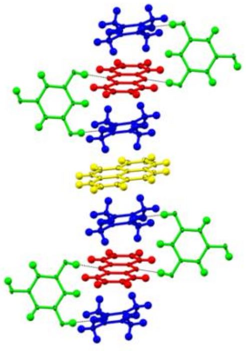

PGL.TMP.PHE.PYR

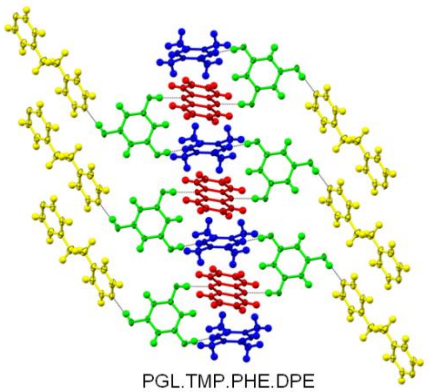

PGL.TMP.PHE.DPE

S4. Crystallographic information

Compound	1	2	3	4	5
CCDC No.	1428091	1428092	1428093	1428094	1428095
Molecular Formula	$C_{38}H_{52}N_6O_4$	$C_{120}H_{160}N_{16}O_{17}$	$C_{36}H_{48}N_6O_6$	$C_{46}H_{44}N_6O_4$	$C_{24}H_{23}N_2O_2$
Formula Weight	124.13	2098.63	660.80	744.87	371.44
Crystal System	Triclinic	Monoclinic	Monoclinic	Triclinic	Triclinic
Space Group	$P\bar{1}$	$C2/c$	$P2_1/c$	$P\bar{1}$	$P\bar{1}$
a (Å)	8.8712(8)	15.533(7)	15.6084(8)	8.983(6)	8.971(4)
b (Å)	10.1819(9)	8.564(4)	13.4786(7)	12.712(9)	10.078(4)
c (Å)	11.2862(10)	21.80(1)	17.2209(9)	17.561(12)	11.666(5)
α (°)	114.633(8)	90	90	89.485(9)	109.665(8)
β (°)	93.749(7)	93.873(7)	90.200(2)	76.291(9)	94.353(7)
γ (°)	101.000(7)	90	90	82.933(10)	90.742(7)
V ($Å^3$)	897.56(16)	2893(2)	3622.9(3)	1933(2)	989.5(7)
ρ_{calc} (g/cm^3)	1.215	1.205	1.212	1.280	1.247
F(000)	354	1128	1416	788	394
μ. (mm^{-1})	0.080	0.081	0.084	0.083	0.080
T (K)	150(2)	150(2)	150(2)	150(2)	150(2)
λ (Å)	0.71073	0.71073	0.71073	0.71073	0.71073
Total Reflns.	9617	14570	109213	19746	9373
Unique Reflns.	4113	3316	7113	8807	4291
Completeness (%)	99.8	99.8	99.9	99.3	99.5
R_{int}	0.020	0.035	0.042	0.051	0.128
R_1 (F^2)	0.0434	0.0513	0.0534	0.0656	0.0953
wR_2(F^2)	0.1240	0.1454	0.2107	0.1919	0.2760
GooF	1.07	1.04	0.85	1.02	0.97
2θ	54	54	52	54	54

Compound	6	7	8	9	10
**CCDC No.**	1428096	1428097	1428098	1428099	1428100
**Molecular Formula**	$C_{23}H_{22}N_3O_2$	$C_{46}H_{50}N_4O_4$	$C_{42}H_{58}N_4O_4$	$C_{38}H_{46}N_4O_4S_2$	$C_{44}H_{40}N_6O_6$
**Formula Weight**	124.13	722.90	682.92	686.93	748.82
**Crystal System**	Triclinic	Triclinic	Monoclinic	Triclinic	Triclinic
**Space Group**	$P\bar{1}$	$P\bar{1}$	$P2_1/c$	$P\bar{1}$	$P\bar{1}$
**a (Å)**	8.778(2)	8.921(5)	11.4579(12)	8.662(2)	9.012(7)
**b (Å)**	10.491(2)	10.723(6)	11.8325(13)	10.289(3)	11.241(8)
**c (Å)**	12.095(3)	10.954(6)	14.9792(16)	11.523(3)	18.355(13)
**α (°)**	66.387(5)	67.417(10)	90	65.003(5)	94.296(13)
**β (°)**	81.548(6)	81.183(9)	107.229(7)	76.930(5)	93.207(11)
**γ (°)**	69.631(5)	78.367(8)	90	80.277(6)	92.248(6)
**V ($Å^3$)**	956.7(4)	944.3(9)	1939.7(4)	903.5(4)	1850(2)
**ρ_{calc} (g/cm^3)**	1.293	1.271	1.169	1.263	1.344
**F(000)**	394	386	740	366	788
**μ. (mm^{-1})**	0.084	0.081	0.075	0.192	0.091
**T (K)**	150(2)	150(2)	150(2)	150(2)	150(2)
**λ (Å)**	0.71073	0.71073	0.71073	0.71073	0.71073
**Total Reflns.**	10145	8881	19976	9411	19286
**Unique Reflns.**	4377	4266	4441	4129	8468
**Completeness (%)**	99.7	98.5	99.9	99.5	98.9
**R_{int}**	0.046	0.050	0.048	0.064	0.116
**R_1 (F^2)**	0.0597	0.0736	0.0551	0.0725	0.0661
**$wR_2(F^2)$**	0.1559	0.2090	0.1532	0.2011	0.2043
**GooF**	1.03	1.07	1.02	1.05	1.07
**2θ**	54	54	54	54	54

Compound	11	12	13	14	15
**CCDC No.**	1428101	1428102	1428103	1428104	1428105
**Molecular Formula**	$C_{56}H_{48}N_8O_6$	$C_{44}H_{46}N_4O_6$	$C_{40}H_{48}N_6O_6$	$C_{29}H_{31}N_3O_2$	$C_{62}H_{54}N_6O_4$
**Formula Weight**	929.02	726.85	708.84	453.57	947.11
**Crystal System**	Triclinic	Monoclinic	Triclinic	Triclinic	Triclinic
**Space Group**	$P\bar{1}$	$P2_1/c$	$P\bar{1}$	$P\bar{1}$	$P\bar{1}$
**a (Å)**	7.576(5)	8.876(4)	8.284(11)	8.9943(15)	9.034(2)
**b (Å)**	9.081(6)	11.941(6)	8.564(12)	9.5169(16)	9.889(2)
**c (Å)**	16.503(11)	17.980(9)	14.60(2)	14.927(3)	14.505(4)
**α (˚)**	96.651(13)	90	78.65(5)	74.162(5)	107.761(8)
**β (˚)**	90.601(11)	97.072(4)	87.26(6)	77.589(5)	101.019(7)
**γ (˚)**	91.433(8)	90	64.40(4)	89.115(6)	91.432(6)
**V ($Å^3$)**	1127.3(13)	1891.2(16)	915(2)	1199.2(4)	1206.5(5)
**ρ_{calc} (g/cm^3)**	1.368	1.276	1.286	1.256	1.304
**F(000)**	488	772	378	484	500
**μ. (mm^{-1})**	0.091	0.085	0.088	0.079	0.082
**T (K)**	150(2)	150(2)	150(2)	150(2)	150(2)
**λ (Å)**	0.71073	0.71073	0.71073	0.71073	0.71073
**Total Reflns.**	12024	17330	8969	12751	12197
**Unique Reflns.**	5147	3710	3974	5486	5504
**Completeness (%)**	99.3	99.8	99.4	99.7	99.4
**R_{int}**	0.106	0.098	0.087	0.036	0.041
**R_1 (F^2)**	0.0562	0.0486	0.0744	0.0555	0.0593
**wR_2(F^2)**	0.1707	0.1530	0.2453	0.1537	0.1634
**GooF**	1.07	1.10	1.14	1.05	1.03
**2θ**	54	52	54	54	54

Compound	16	17	18	19
**CCDC No.**	1428106	1428107	1428108	1428090
**Molecular Formula**	$C_{64}H_{56}N_4O_4$	$C_{54}H_{54}N_6O_6$	$C_{56}H_{56}N_6O_8$	$C_{44}H_{44}N_6O_6$
**Formula Weight**	124.13	883.03	941.07	752.85
**Crystal System**	Triclinic	Triclinic	Triclinic	Triclinic
**Space Group**	$P\bar{1}$	$P\bar{1}$	$P\bar{1}$	$P\bar{1}$
**a (Å)**	9.0005(8)	8.6332(13)	10.1510(11)	7.812(9)
**b (Å)**	10.0381(9)	9.0194(14)	11.2084(12)	8.763(10)
**c (Å)**	14.4894(13)	14.869(2)	12.3363(13)	14.300(16)
**α (°)**	108.432(8)	105.200(4)	102.012(3)	95.009(14)
**β (°)**	101.780(7)	91.263(4)	105.153(3)	95.415(11)
**γ (°)**	90.484(6)	91.519(4)	112.368(3)	101.04(2)
**V ($Å^3$)**	1212.0(2)	1116.4(3)	1176.1(2)	951.0(19)
**ρ_{calc} (g/cm^3)**	1.295	1.313	1.329	1.315
**F(000)**	500	468	498	398
**μ. (mm^{-1})**	0.081	0.087	0.090	0.089
**T (K)**	150(2)	150(2)	150(2)	150(2)
**λ (Å)**	0.71073	0.71073	0.71073	0.71073
**Total Reflns.**	12950	29237	42928	10088
**Unique Reflns.**	5546	4848	4600	4348
**Completeness (%)**	99.8	99.8	99.4	98.8
**R_{int}**	0.028	0.095	0.036	0.066
**R_1 (F^2)**	0.0502	0.0881	0.1045	0.0518
**wR_2(F^2)**	0.1376	0.2749	0.4122	0.1708
**GooF**	1.03	0.99	1.19	1.11
**2θ**	54	54	52	54
